# The complete mitochondrial genomes of two scuttle flies, *Megaselia spiracularis* and *Dohrniphora cornuta* (Diptera: Phoridae)

**DOI:** 10.1080/23802359.2020.1730256

**Published:** 2020-02-27

**Authors:** Dianxing Feng, Jieqin Li, Guangchun Liu

**Affiliations:** a College of Life Science and Engineering, Shenyang University, Shenyang, Liaoning, China;; b College of Agriculture, Anhui Science and Technology University, Fengyang, Anhui, China

**Keywords:** *Megaselia spiracularis*, *Dohrniphora cornuta*, mitochondrial genome, Phoridae

## Abstract

*Megaselia spiracularis* and *Dohrniphora cornuta* were two forensically important flies in relatively sealed environments. Their mitochondrial genomes were first sequenced, annotated, and phylogenetic analyses were performed with other 8 species of the Asehiza in this study. Maximum-likelihood phylogenetic tree revealed that Phoridae is closer to Platypezidae and Lonchopteridae within Diptera. This work increases the databases of Phoridae species, and contributes to the further study of species identification and phylogenetics of this family.

Due to their tiny size, *Megaselia spiracularis* and *Dohrniphora cornuta* were reported as forensically important flies in relatively sealed environments (Thevan et al. 2010; Disney et al. 2014). The mitochondrial genome was often used for the research of species identification and phylogenetic relationships (Fu et al. 2016). In this study, the complete mitogenome of *M. spiracularis* was 16,035 bp and showed 78.4% of AT. The mitochondrial genome of *D. cornuta* was 15,849 bp and had 78.4% of AT. Their circular genomes contained 13 protein-coding genes, 22 transfer RNA genes, 2 ribosomal RNA genes, and a non-coding AT-rich region located between 12S rRNA and tRNA*^Ile^*, which was 1192 bp for *M. spiracularis* and 997 bp for *D. cornuta*.

*Megaselia spiracularis* (voucher SYU-FD-PHO002) and *D. cornuta* (voucher SYU-FD-PHO003) were collected in Shenyang (41°48′ N, 123°25′ E), Liaoning province, China. Each specimen was identified by entomologist according to taxonomic keys (Liu 2001). The voucher specimens and their DNA were deposited in the Natural History Museum of Shenyang University. Total DNA was extracted using a DNeasy Blood & Tissue Kit (Qiagen, Hilden, Germany) following the manufacturer protocol. The mitogenomes of these two flies were amplified in eight overlapping fragments (Nelson et al. 2012). Within each long PCR product, the full double-stranded sequence was determined by primer walking. DNA fragments were sequenced on both strands by the commercial corporation (GenScript Co. Ltd., Nanjing, China). Their mitogenomes have been submitted to GenBank with accession numbers MN832848 and MN832849.

Phylogenetic analyses of the two phorid flies with other eight species from the Asehiza were performed with two species, *Chrysomya megacephala* and *Ravinia pernix* from the Schizophora as the outgroup (Figure 1), which showed the Phoridae is closely related to the Platypezidae and Lonchopteridae, and they are subordinate to Platypezoidea. The two superfamilies, Platypezoidea and Syrphodidea including Syrphidae and Pipunculidae constitute the Asehiza. The ML phylogenetic tree supported the system proposed by McAlpine about the taxonomic position of Phoridae within Diptera (McAlpine and Wood 1989). In addition, the mitochondrial genome sequences could also be useful in identifying Phoridae species with forensic importance.

**Figure 1.**
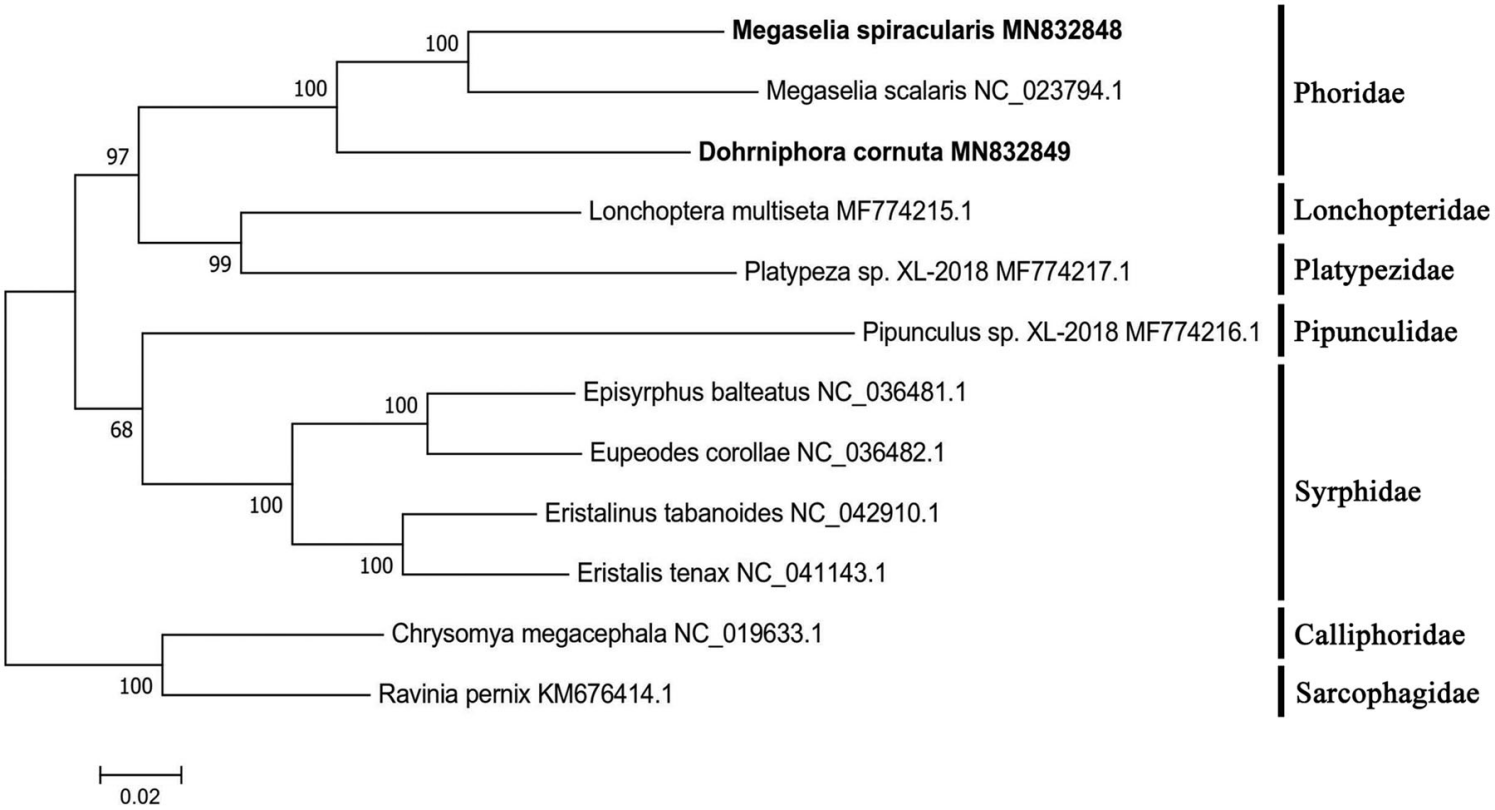
ML phylogenetic tree was constructed based on mitogenome sequences of 10 flies from the Asehiza and two flies from the Schizophora as the outgroup using MEGA 7 software. The numbers in the nodes indicated the support values with 1000 bootstrap replicates.
